# Restoring natural upper limb movement through a wrist prosthetic module for partial hand amputees

**DOI:** 10.1186/s12984-023-01259-9

**Published:** 2023-10-05

**Authors:** Seoyoung Choi, Wonwoo Cho, Keehoon Kim

**Affiliations:** 1 Department of Mechanical Engineering, POSTECH, Pohang University of Science and Technology, Gyeongbuk, 37673 Republic of Korea; 2 Present Address: Hyundai Rotem Company, Uiwang-si, Gyeonggi-do Republic of Korea; 3 Institute for Convergence Research and Education in Advanced Technology, Yonsei University, 50 Yonsei-ro, Seoul, 03722 Republic of Korea

**Keywords:** Partial hand amputation, Wrist rotation, Muscle synergy, Double parallelogram mechanism, Upper-limb movement, NMF, Motion analysis, Hand function

## Abstract

**Background:**

Most partial hand amputees experience limited wrist movement. The limited rotational wrist movement deteriorates natural upper limb system related to hand use and the usability of the prosthetic hand, which may cause secondary damage to the musculoskeletal system due to overuse of the upper limb affected by repetitive compensatory movement patterns. Nevertheless, partial hand prosthetics, in common, have only been proposed without rotational wrist movement because patients have various hand shapes, and a prosthetic hand should be attached to a narrow space.

**Methods:**

We hypothesized that partial hand amputees, when using a prosthetic hand with a wrist rotation module, would achieve natural upper limb movement muscle synergy and motion analysis comparable to a control group. To validate the proposed prototype design with the wrist rotation module and verify our hypothesis, we compared a control group with partial hand amputees wearing hand prostheses, both with and without the wrist rotation module prototype. The study contained muscle synergy analysis through non-negative matrix factorization (NMF) using surface electromyography (sEMG) and motion analyses employing a motion capture system during the reach-to-grasp task. Additionally, we assessed the usability of the prototype design for partial hand amputees using the Jebsen-Taylor hand function test (JHFT).

**Results:**

The results showed that the number of muscle synergies identified through NMF remained consistent at 3 for both the control group and amputees using a hand prosthesis with a wrist rotation module. In the motion analysis, a statistically significant difference was observed between the control group and the prosthetic hand without the wrist rotation module, indicating the presence of compensatory movements when utilizing a prosthetic hand lacking this module. Furthermore, among the amputees, the JHFT demonstrated a greater improvement in total score when using the prosthetic hand equipped with a wrist rotation module compared to the prosthetic hand without this module.

**Conclusion:**

In conclusion, integrating a wrist rotation module in prosthetic hand designs for partial hand amputees restores natural upper limb movement patterns, reduces compensatory movements, and prevent the secondary musculoskeletal. This highlights the importance of this module in enhancing overall functionality and quality of life.

## Background

Partial hand amputees are the most prevalent, accounting for almost 90% of upper limb amputations [1, 2]. Partial hand amputation is the loss of a part of the hand due to a disease or accident, which causes difficulties in activities of daily living (ADL) [2]. To overcome the limitations in ADL due to hand amputation, personalized prosthetic hands have been developed to fit the shape and function of the missing hand.

Prosthetic hands have been developed for research and commercial use as cosmetic or passive [3–8], myoelectric [9–11], electrically powered [12–15], cable-controlled [16, 17], and 3D-printed [18] hands depending on the designing and operating method. Prosthetic hand designs, in particular, have been proposed to mimic the complex hand architecture of the human hand, which has the highest degrees of freedom (DOF) and hand dexterity [15, 19, 20].

However, despite the development of prosthetic hands, the usability of partial hand/hand amputees has been declining. Approximately 35% of partial hand amputees who undergo reconstructive surgery experience limitations in wrist movement, resulting in secondary musculoskeletal damage, discomfort, and pain from repetitive compensatory movements [21–24]. The prosthetic hand is closely related to wrist movement owing to the anatomical features of the hand and wrist [25]. The wrist joint enables the hand to move in the appropriate direction to achieve the final goal with minimal control of the upper limb [26, 27]. In particular, the rotational movement of the wrist (pronation/supination) is important for controlling hand orientation [23, 28, 29].

Despite the importance of the wrist in upper limb movement related to the hand function, prosthetic wrist design has received less attention and has been less developed compared to hand prosthetics. The wrist has three DOFs [29]. These DOFs influence the design of wrist prosthetics [30–33], allowing them to be implemented as serial, parallel, or hybrid mechanisms based on the kinematic configuration of the hand or terminal device. Most wrist prosthetics [32] were designed to rely on manual control to adjust the direction of the hand, primarily to maintain a simple system and avoid adding weight and length that could fatigue the user. Recent research [14, 30, 31, 34, 35] has advanced the development of actively operated wrists powered by electric motors, pneumatics, or hydraulics. Currently, commercial products [36] from companies like Ottobock, Touch Bionics, and Shanghai Kesheng Prosthesis are the most renowned. However, the majority of previous prosthetic hands, including wrist function, were designed for use with relatively consistent amputated stumps, such as trans-humeral, trans-radial, and wrist disarticulation amputations.

Prosthetic hands for partial amputation have been reported to be divided into a modular finger and a wrist-powered finger prosthesis [14]. The modular finger prosthesis includes 3D-printed Knick Finger, Naked Finger, M-finger, and S-finger, which only cover the finger part [14, 36–40]. The aforementioned prosthetic hands have a disadvantage in that they are designed to allow only hand movements and, therefore, cannot accommodate limited wrist movements. The design of wrist prostheses for individuals with partial hand amputation is limited by several factors [14, 39].

Modular prostheses with wrist rotation designs are lacking for individuals with partial hand amputations due to (1) design application space is limited since the amputation stump is inconsistent owing to anatomical diversity and (2) miniaturization is difficult due to relatively high cost and functional requirements for wrist rotation movement. Hence, no studies have proposed modular prostheses with wrist rotation designs applicable to individuals with inconsistent amputated stumps, such as those with partial hand amputations.

Several studies have reported that incorporating wrist movement in prosthetic hands improves hand function and dexterity or upper extremity movement [28, 41]. According to [28], the multiple DOF hand/1DOF wrist use and the single DOF hand (hand open/closed)/2DOF wrist use showed that both configurations showed close-to-high performance in a common task. In addition, other studies [29, 42] have shown that upper-limb amputees who use a prosthesis lacking wrist rotation movement adopt a compensatory movement strategy while performing goal-oriented tasks or reach-to-grasp actions. However, these studies used simulations or emulated architecture to validate healthy subjects rather than actual amputees using prosthetics.

Further evaluation is required to assess the usability and recovery level of upper limb movement when wrist rotation is allowed for actual partial hand amputees. The neuromuscular strategy for natural movement is designed to enable optimal performance of a particular task with minimal energy expenditure and without the need for compensatory patterns, as in typical motor development processes [43, 44]. It has been reported that most healthy people generate similar movement patterns while performing the specific task as reach-to-grasp [42, 45, 46]. Conversely, compensatory patterns are alternative neuromuscular strategies that the body uses when a naturally prescribed neuromuscular strategy is no longer viable for producing a given movement. The neuromuscular strategy for generating movement can be determined according to the activity level of the muscles involved in the motion. This information can be used for muscle synergy and kinematic analyses.

Muscular synergy simplifies complex problems by modularizing multiple muscles, resulting in a multi-joint musculoskeletal system of displacements in the working space between the initial position of the hand and the target of movement. This system is part of the upper limbs’ multidimensional musculoskeletal system [47, 48]. Compensatory movements, resulting from limited joint mobility, can change the strategy of muscle synergy in patients with stroke or those with musculoskeletal pain. However, there have been no studies conducted on amputees.

Repetitive compensatory movements in the upper limb due to limited wrist rotational movements in amputees can cause secondary damage and pain to the musculoskeletal system due to unbalanced use, such as underuse or overuse. To provide natural upper-limb movement, prevent secondary injuries, and reduce the dropout rate during prosthetic use, it is necessary to investigate whether the wrist rotation motion affects muscle synergy and the upper-limb movement pattern strategy in patients with partial hand amputation [28, 42, 49, 50].

This study aimed to propose a design of a prosthetic hand with a wrist rotation module prototype applicable to patients with partial hand amputation and to investigate the effect of design on upper limb movements and the muscle synergies by comparing the reach-to-grasp motion between a control group and an amputee. For verification, a control group including ten healthy subjects and a partial hand amputee was divided into two cases: when only the prosthetic hand (without pronation/supination (P/S)) was worn, and when the prosthetic hand and wrist rotation module (with P/S) were used. We evaluated the usability of the prosthetic hand with a wrist rotation module using the Jebsen–Taylor hand function test (JHFT) for partial hand amputees (intact, without P/S, and with P/S). In addition, during the reach-to-grasp task [51], motion analysis was performed using the trunk, shoulder, elbow, and wrist joint angles. The muscle synergy was analyzed for 14 muscles in the upper limb using the NMF (Non-negative Matrix Factorization) technique [52], it is commonly used for muscle synergy and decomposes the muscle activity of multichannel muscles to quantify temporal and spatial coordination analysis. We identified the movement strategies used to complete the task based on muscle synergy. In addition, the control group was compared with two cases (with and without P/S) of partial hand amputation.

The summary of the contribution points of this study is as follows.

1. We designed the wrist rotation module for a prosthetic hand which is applicable to a partial hand amputation.
2. Analysis of the effect of the wrist rotation module through quantitative motion analysis and muscle synergy of the subject's upper limb with partial hand amputation and the control group.

## Methods

In section “Wrist rotation module prototype design” describes a previously developed prosthetic hand (Re-fill [40, 53]) and a wrist-rotation module prototype design applicable to subjects with a partial hand amputation. In section “Experiments for validation” consists of two experiments. Experiment 1 was conducted to verify the usability of the ADL of the proposed prosthetic hand using the wrist rotation module method, and a JHFT was conducted on an amputee subject. In addition, to validate the upper limb movement effectivity, we performed a reach-to-grasp task in Experiment 2, which represents the fundamental upper limb movement related to hand function. Here the muscle synergy and related motion analyses were compared between a control group and partial hand amputees with and without P/S during the reach-to-grasp task. Finally, Sect. “Data analysis and statistics” explains the data analysis and statistical methods used in the experiment in Sect. “Experiments for validation”.

### Wrist rotation module prototype design

#### Previous work: Re-fill project

**Fig. 1:**
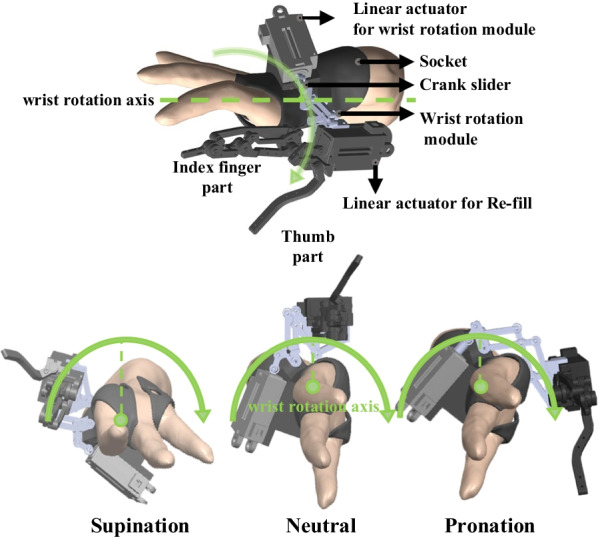
Description of the proposed wrist rotation module prototype and the Re-fill (proposed prosthetic hand), and its wrist rotation motion

The common prosthetic hand could be customized by determining the shape and function of the amputee's remaining hand [19]. Our team developed and customized a prosthetic hand called a Re-fill for a partial hand amputee (Table 1) [40, 53]. The Re-fill consisted of the thumb and index finger parts (Fig. 1). The thumb and index finger are the passive and active joints, respectively. The index finger can be mainly driven by one linear actuator with 3DOF like the human index finger: distal, proximal interphalangeal, and metacarpophalangeal joints of the human index finger. Functionally, adaptation to the shape of an object is possible. Re-fill can perform hand grasping, holding, and releasing by opening and closing, which account for more than 70% of hand functions [26]. In addition, the straps could be worn easily and comfortably. Using the box and block test, the Re-fill validated its performance in moving eight blocks in 60 s.

**Table 1:** Characteristics of a partial hand amputee subject

Character	Subject
Year/gender	51 y/ male
Height/weight	173 cm/ 68 kg
Job	Electrical engineer
Onset date	2012
Cause of amputation	Car accident
Amputated side	Right side Thumb phalanxes, 2nd phalanxes, 1st, 2nd metacarpus, trapezium bone
Residual range of motion	Shoulder/elbow: full flexion/extension Wrist: Flexion/extension 60 deg/40 deg, Supination/pronation 36 deg/30 deg Hand: N/A
Contracture	Wrist flexion 55 deg, Hand flexion

However, the amputee has been undergoing limited wrist rotation and reduced hand movement due to long-term internal plate fixation in both the fractured radius and ulnar bones after orthopedic surgery. He could flex or extend his wrist within a limited range of rotation. Therefore, we should consider designing a wrist-rotation module for enhanced hand function and efficient upper-limb movements without causing secondary damage. The characteristics of the amputee subjects (Table 1) were acquired to design a prosthetic hand and a wrist-rotation module applicable to the amputation hand.

Table 1. Characteristics of a Partial Hand Amputee Subject.

#### Specification for proposed wrist rotation module prototype

We proposed a wrist rotation module prototype for a partial hand amputee. The wrist-rotation module consisted of pronation and supination along the longitudinal axis of the anatomical transverse plane. We aimed to meet the two conditions for the wrist rotation module design for a partial hand amputee as mentioned in Sect. “Background”: (1) the prototype module’s axis of wrist rotation on the partial hand amputee allows the anatomical axis of the wrist rotation to be followed without interfering with the hand and the Re-fill, (2) low cost, small size, and design according to the required wrist rotation function (Table 2) [35].

**Table 2:** Required functions of proposed prototype

Dimensions	Required functions	Proposed
Hand (thumb and index finger) and wrist joint [26]		
Weight (g)	540	500
Wrist thickness (mm)	43.0	44.0
Wrist width (mm)	63.1	78.2
Wrist circumference (mm)	172.4	201.2

Degree of freedom	Functional ROM [35]	Proposed
Wrist pronation (deg.)	65	70
Wrist supination (deg.)	77	70
Hand open (deg.)	50	90
Hand close (deg.)	70	90

Degree of freedom	Torque: mean [35]	Proposed
Wrist pronation (N)	9.0	12.0
Wrist supination (N)	9.5	7.0
Hand open/close (N)	5.0	4.6

Degree of freedom	Speed [35]	Proposed
Wrist pronation/ supination (rad/s)	1.7	1.7
Hand open/ close (rad/s)	1.7	1.7

To satisfy the first condition for the wrist rotation module prototype design, it is important to know the anatomical structure of the wrist and forearm to determine the wrist rotation axis. Wrist rotation involves the distal radius wrapping around the ulna as the proximal radial head spins. Therefore, wrist rotation begins in the forearm (radial head), is transmitted to the wrist adjacent to the radius, and has a virtual axis centered on the third finger, based on the hand’s anatomy [26, 27, 54]. As a result, the thumb and index fingers produce a circular motion as the wrist rotates. Therefore, we applied a double parallelogram mechanism (DPM) to design a wrist-rotation module with a virtual axis aligned with the longitudinal axis [6, 55]. The DPM can move in a semicircular shape, similar to wrist rotation. A remote center (RC) can be formed at the center of the wrist to rotate the prosthetic finger without colliding with the residual hand. The wrist joint rotates along one axis based on the third finger and draws a semicircle based on the thumb (Fig. 1).

We confirmed functional range of motion (ROM), force, and speed for rotational motions in the wrist (the pronation and supination movements) to meet the second conditions in the proposed wrist rotation module in Table 2. The requirements for the wrist rotation module design were defined by human anthropometric data and the functional needs of activities of daily livings (ADLs), referencing previous research [26]. In Table 2, the first row, labeled 'Hand (thumb and index finger) and wrist joints,' was derived from anthropometric data by taking into account the height and weight of the participating amputees (Table 1) [26]. For the second to fourth rows, the 'Function ROM', 'Torque', and 'Speed' items, we chosen the information on the joint angles, forces, and speeds required for the ADLs as suggested by prior research [35]. It was made at a low cost and lightweight (500 g) to satisfy these conditions. Because a non-back-drivability mechanism is an essential requirement for prostheses [57], a lead-screw-based linear actuator (PLS-5030, Potenit Inc., Korea) was selected, and a slider-crank structure was used in consideration of space efficiency. We provided detailed information on slider crank systems in [40, 53].

The amputee is able to perform full supination motion and can rotate the wrist up to the neutral position (wrist rotation 0 deg), but full pronation is not possible. In the case of full pronation, the radius moves horizontally over the ulna, causing rotation of the longitudinal axis of the upper limb. Therefore, the RC axis to be created by the mechanism was determined to rotate based on the third finger of the amputee’s 3D-scanned hand model so that the DPM could perform supination and pronation movements.

#### Description of wrist rotation module prototype

**Fig. 2:**
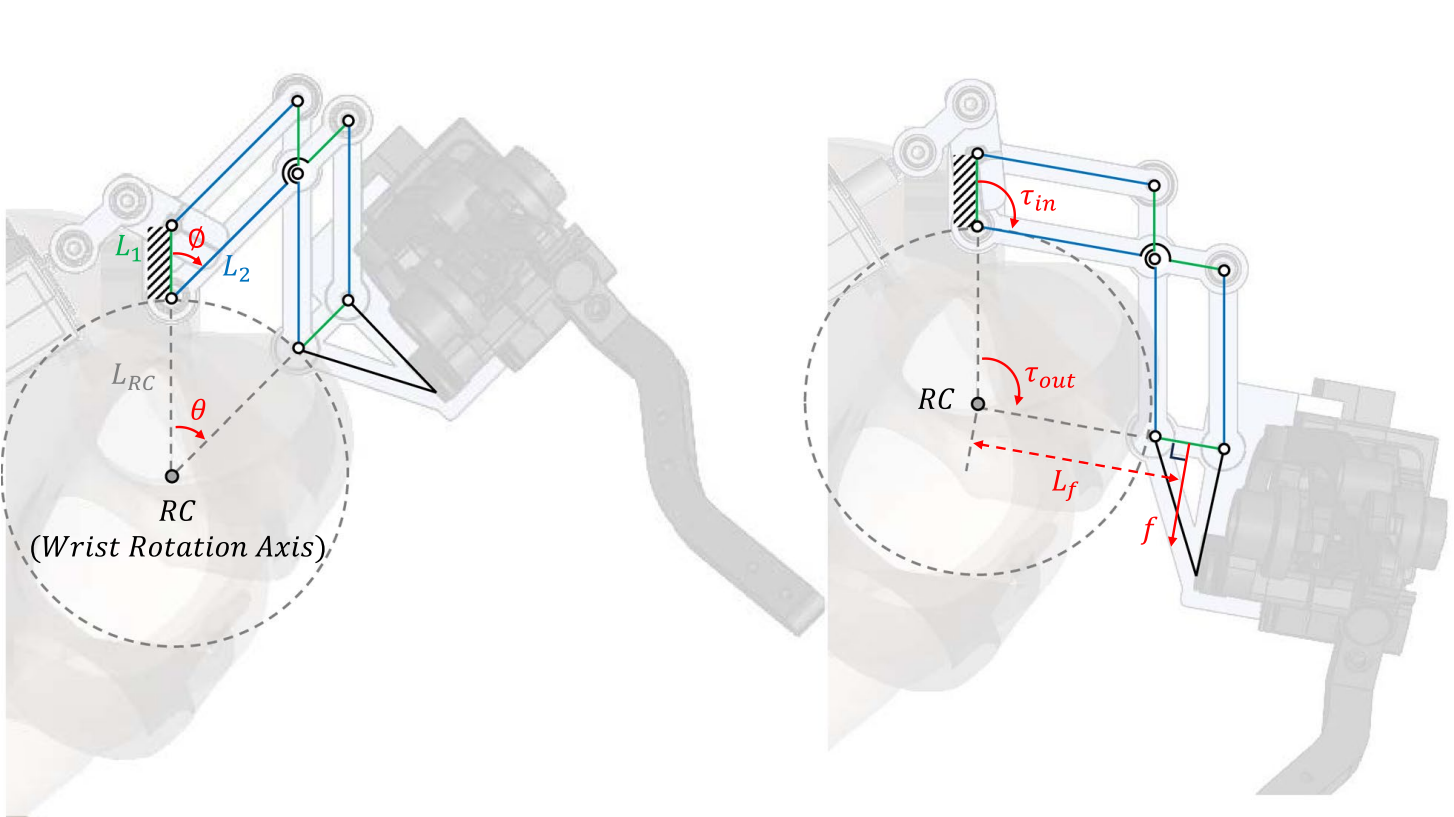
The kinematics of the proposed mechanism for wrist rotation module prototype

The kinematics of the proposed mechanism is formulated based on parallelograms (Fig. 2). In order to select the location of RC, which is the axis of wrist rotation, the main link lengths were determined only ${L}_{1}$ and ${L}_{2}$ illustrated by the green line and blue line. As a result, DPM consisted of a pair of parallelograms of equal size and shape, forming a parallelogram with the RC as a defined vertex and all four sides of length ${L}_{RC}$. Following the shape changing parallelogram, ${L}_{RC}$, a rotation radius of DPM, is as follows.

1 $${L}_{RC}={L}_{2}$$

We measured the length from the wrist rotation axis to the furthest point of amputee’s residual hand to evade the collision between the DPM and the hand. As a result, the length of ${L}_{RC}$ which is same as ${L}_{2},$ and ${L}_{1}$ was set to 25 mm and 10 mm to satisfy the small size of mechanism which can rotate without interfering with the hand.

According to these parameters, the angle of wrist rotation $\theta$ has the same degree with $\varnothing$, the angle between ${L}_{1}$ and ${L}_{2}$, which is an input angle of the crank slider. Therefore, the relation between $\varnothing$ and $\theta$ was obtained as follows:

2 $$\theta =\varnothing$$

3 $$\dot{\theta }=\dot{\varnothing }$$

4 $${\tau }_{out}={\tau }_{in}$$

$\dot{\varnothing }$ and $\dot{\theta }$ are the angular velocity of the crank input and the wrist rotation. ${\tau }_{in}$ and ${\tau }_{out}$ are the torque from the crank input and the wrist rotation.

Since, the force of wrist rotation from supination and pronation was generated by ${\tau }_{out}$, the force $f$ which pushed the coupler in normal direction at the middle of link, can be obtained as follows

5 $${L}_{f}={L}_{RC}+\frac{{L}_{1}}{2}$$

6 $$f=\frac{{\tau }_{in}}{{L}_{f}}$$

${L}_{f}$ is the length of moment arm of ${\tau }_{out}$. ${\tau }_{in}$ was calculated in our previous work [40] as 0.3 Nm. As a result, $f$ is 10N which satisfy the required force of wrist supination and pronation.

**Fig. 3:**
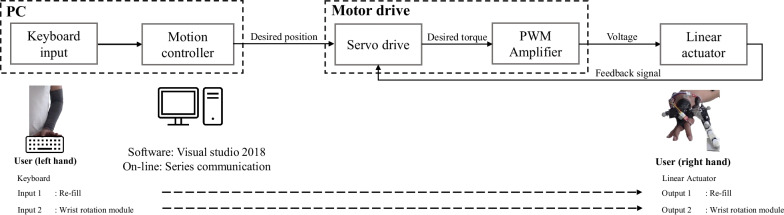
Operating and control principles for wrist rotation module and Re-fill

Through keyboard-input and a motion controller, we assign desired positions to the motor drive for the Re-fill and wrist rotation module, respectively (Fig. 3). The servo drive then calculates the torque required to manipulate the Re-fill and wrist rotation module, which is a position controller, and PWM amplifier provides the corresponding voltage to the motors in order to operate the prosthesis. In our previous research, our team developed the Re-fill [40] using myoelectrical input method. However, in this study, we opted for the more intuitive keyboard input method because the EMG-driven input approach could potentially influence that the muscle synergy patterns in the participated amputee [43].

### Experiments for validation

#### Participants

We recruited ten able-bodied right-handed subjects (8 males and 2 females) with a mean age of 25.5 ± 1.2 years, height of 174.3 ± 3.9 cm, and weight of 67.4 ± 10.8 kg, as well as one individual with a partially amputated hand (Table 1) to compare the effect of upper limb movement (muscle synergy and movement patterns). The inclusion criteria were as follows: (1) no orthopedic surgery or disease in the right upper extremity, (2) no neurological damage, and (3) no pain or abnormal sensations in the right upper extremity or hand. Participants voluntarily consented to participate in the experiment after receiving an explanation of its contents and procedures. This study was approved by the POSTECH Institutional Review Board (No. PIRB-2022-E012).

#### Experiment 1: Jebsen-Taylor hand function test

We used the Jebsen-Taylor hand function test (JHFT), a widely used assessment tool that measures a broad range of the uni-hand functions for ADL [51]. The JHFT consists of seven subsets: writing, simulated page-turning, lifting small objects, simulated feeding, stacking, and lifting large, lightweight, and heavy objects. The test is scored based on the time it takes to complete the task (units: seconds, maximum 120 s) and reflects speed rather than the performance quality. The JHFT allows compensatory movement of the trunk and shoulders during each task.

**Fig. 4:**
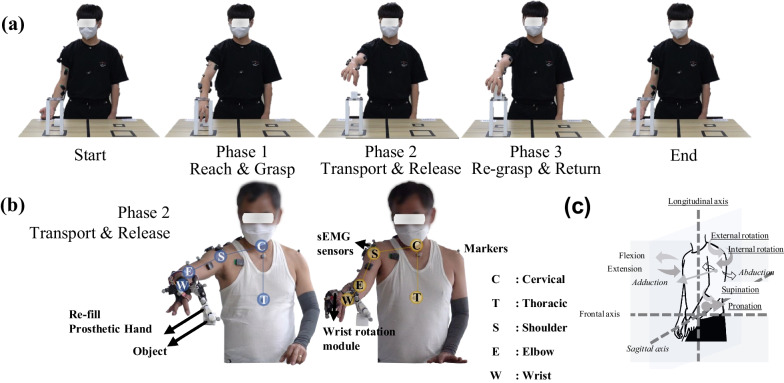
Description of the reach-to-grasp task. **a** All phases of the motions the reach-to-grasp task in the control group. **b** In phase 2, the partial hand amputee used only the Re-fill without P/S (left side), and a partial hand amputee used the Re-fill with P/S (right side). **c** Anatomical plane and defined joint motions

We prepared a standardized JHFT tool, including a pen and paper to record scores, a stopwatch or timer, and the JHFT manual. The participants sat comfortably and had their tested hands positioned on a table. We explained the test's purpose to the recruited amputee and provided instructions for each subset. According to the instructions, each subset was tested quickly and accurately. The examiner used a stopwatch to measure the time required to complete each task, and the scores were recorded. For consistency in the test, we requested that the subject maintain a seated posture while performing the tasks and asked whether they were permitted to make compensatory movements of their trunk and shoulders. Even then, we recorded 120 s for the tasks that he could not complete. The JHFT was repeated twice, and the fastest test was chosen. The test proceeded in the following order: the subject’s intact hand (left hand), an amputated hand (right hand) with only Re-fill without P/S, and finally an amputated hand (right hand) with Re-fill and with P/S (Fig. 4b).

#### Experiment 2: reach-to-grasp task

The reach-to-grasp task (Fig. 4), including reach, grasp, transport, release, and return, can be defined as the most fundamental movement performed by the upper limbs [45]. We simultaneously investigated the muscle synergy and movement patterns in the upper limb according to the prosthetic hand with and without P/S in one person with a partially amputated hand during the reach-to-grasp task (Fig. 4b). To validate this, we compared a control group including 10 healthy subjects with muscle synergy and movement patterns while performing a reach-to-grasp task (Fig. 4a).

The reach-to-grasp task (Fig. 4) involved moving an object from the desk on which the participant was standing to the shelf on the same desk. For a consistent experimental environment, the desk's height was adjusted to be positioned in front of the pelvis (anterior superior iliac spine), considering the participant’s leg length. The position of the object on the desk and that on the shelf were fixed at designated locations. The object was placed on the sagittal plane of each arm approximately 10 cm from the subject.

The task can be divided into three phases (Fig. 4a) [43, 45]. Phase 1 involves reaching and grasping an object placed at a designated location on the table. Phase 2 requires transporting the picked object and releasing it onto the shelf on the table. Phase 3 engages regrasping the object and returning it to the table. All participants took the initial posture before Phase 1 and returned to the initial posture after Phase 3. Prior to initiating the motion, the initial posture was as follows: 0 degrees of the shoulder, 90 degrees of the elbow, and 90 degrees of wrist supination. The reach-to-grasp task was performed six times per set and repeated in four sets. At the end of each set, all subjects rested for at least 30 s. All the participants performed the reach-to-grasp motion 24 times under the guidance of an instructor. Participants completed the task to familiarize themselves with it before the experiment and performed it consistently. During the reach-to-grasp motion, participants were allowed to perform the movements as naturally as possible, and the initial posture at the beginning of the motion was maintained.

To analyze the muscle synergy using NMF [52, 58, 59], the surface electromyography (sEMG) sensors (Delsys Trigno EMG, Delsys, MA, USA) were attached to the muscles correlated in the reach-to-grasp task. We chose muscles based on two criteria: muscles used during the reach-to-grasp task [43, 58, 60–63] and muscles measurable by the amputee participating in our study. sEMG sensors were recorded for 14 muscles on the upper limb [63], which are the anterior deltoid (ADEL), posterior deltoid (PDEL), middle deltoid (MDEL), supraspinatus (SUFR), latissimus dorsi (LATD), pectoralis major (PECT), teres minor (TERE), infraspinatus (INFRA), biceps brachii (BIC), triceps brachii (TRI), pronator teres (PRO), supinator (SUPI), extensor digitorum (WEX), and flexor digitorum (WFLE). We followed SENIAM recommendations [64] for skin preparation and electrode placement [65]. Before performing the task, all participants completed a maximal voluntary contraction (MVC) test for each muscle to normalize the sEMG signal. A single clinically experienced examiner performed this test on all subjects to ensure the consistency of measurements. During the test of each muscle, the subject was asked to sit and position the arm for each muscle according to the examiner's instructions. All participants performed MVC five times on each muscle as performed by the examiner, with 30 s of rest between each contraction to prevent muscle fatigue.

Simultaneously, we acquired the kinematic data using an eight-camera motion capture system (VICON, Oxford Metrics Ltd., Oxford, UK) to analyze the upper limb movement patterns during the task. The motion capture system was acquired at 125 Hz. We targeted the shoulder, elbow, wrist joints, and the trunk to monitor upper limb kinematics. The set of markers is defined in the "Upper body modeling with Plug-in Gait" model provided by the motion capture system. The model utilized 17 reflective markers placed at anatomical locations [66–69]. Ten markers are attached to the torso [66]. Seven markers were attached to the right shoulder on the acromioclavicular joint, upper arm on the lower lateral 1/3 surface, lateral condylar of the elbow, lower arm on the lower lateral 1/3 surface, lateral/medial sides on the wrist, and 2nd finger. On the amputated hand wearing the prosthesis, two wrist markers (lateral and medial wrists) and an index finger marker were placed in the same position as the actual wrist joint. To obtain the angle, marker sets were attached following the guidelines of this model, and subsequently, the markers were captured using the system. After capturing, the joint angles were post-processed using the VICON NEXUS software (VICON, Oxford Metrics Ltd., Oxford, UK) based on the captured markers.

### Data analysis and statistics

#### JHFT score

The JHFT is scored by measuring the completion time for each of the seven tasks. The subtest score equaled the number of seconds required to complete the task, and the maximum score for each subtest was 120. The total score was the sum of the scores from all subtests calculated separately for each hand. The lower the score, the better the participant’s hand function. We compared the JHFT scores between the intact side, the amputated side with only the Re-fill with P/S, and the amputated side without P/S. We compared the standardized JHFT scores (healthy people dominant/non-dominant hands) for age-specific healthy males [70].

#### Reach-to-grasp task for analysis of muscle synergy and its motion analysis

First, we analyzed the muscle synergy using NMF [59]. The sEMG signals were collected at 1000 Hz. We normalized the time from the start to the finish of the task (0 = start, 1 = finish). The following preprocessing steps were performed: band-pass filtering with a cut-off range of 20–450 Hz, notch filtering (cut-off: 60 Hz), rectification, low-pass filtering with a cut-off frequency of 2 Hz, and subtraction of the average when no movement occurred before the starting position. After sEMG preprocessing and subsequent data removal, the muscle synergy was extracted by applying the NMF algorithm. The NMF consists of the decomposition of multi-muscle sEMG signals into two matrices representing spatial muscle synergies (W, weighted muscle coefficient) and temporal muscle synergies (C, muscle activation pattern) [59]. Therefore, the decomposition of the sEMG signal into two matrices represents the control modules for the movement patterns, which are encoded in terms of the spatiotemporal neuromuscular strategy employed until the task is completed. The factorization of muscle activity is expressed as follows:

7 $${{\varvec{E}}}_{n\times t}={{\varvec{W}}}_{n\times m}\times {{\varvec{C}}}_{m\times t}+{\varvec{e}},$$

where n is the number of muscles, and t is the number of time points. The initial matrix consisted of normalized sEMG data and the average of three cycles for each of the 14 muscles. E is a 14 × 501 matrix, W represents an n × m matrix, and m is the number of synergies and represents the muscle synergy. C is an m × t matrix that represents the activation coefficient, and e is the residual error matrix. For each subject, we repeated the analysis by varying the number of synergies between 1 and 14 and selected the least number of synergies fulfilling the global variance accounted for (gVAF > 90%) and VAF for each muscle (mVAF > 75%). VAF is 100% the coefficient of determination from the uncentered Pearson correlation coefficient [58, 71].

The upper limb movements during the reach-to-grasp task were evaluated and compared with the control group’s upper limb movements with and without wrist prostheses (P/S) in amputees. The trunk flexion/extension, trunk rotation, shoulder flexion/extension, shoulder abduction/adduction, shoulder internal/external rotation, elbow flexion/extension, wrist flexion/extension, and wrist pronation/supination were also assessed (Fig. 4c).

After obtaining the joint angles derived by Eular angles in VICON NEXUS, we redefined the angles to understand the changes in the joints during the task. We subtracted all calculated joint angles based on the defined initial posture. All participants were instructed to maintain this initial posture at the beginning. It as follows:

8 $${{\varvec{\theta}}}_{j}={{\varvec{\theta}}}_{j\_m}-{\theta }_{j\_i}$$

$\theta$ is the joint angles. $j$ represents each joint, $m$ denotes the joint angles calculated across all task duration using VICON NEXUS. $i$ denotes the initial value.

During the reach-to-grasp for each joint, we confirmed the angle change for each joint and compared and analyzed the angle change for the task. In addition, we aimed to quantify the compensatory movement using the joint angles of the control group subjects and amputee patients with and without P/S. Compensatory movement (CM) refers to a typical pattern that compensates for the loss of mobility in one part of the body. This is achieved by either underusing or overusing other parts of the body to achieve a final goal. To quantify the CM, we used normalization through the average difference between the maximum and minimum values ($RO{M}_{n}$) of each joint angle in all control group subjects of the control group during reach-to-grasp task. Then, the average value of each joint angle in the control group was subtracted from the average value of the body segment angles for subjects with and without the wrist rotation module configuration and each trial as follows:

9 $$CM=\frac{\left|{\alpha }_{w}{- \sigma }_{n}\right|}{{ROM}_{n}},$$

where CM is the ratio of compensatory movement [28], $RO{M}_{n}$ is the average ROM of all control group subjects in each α and $n$ is the joint. ${\sigma }_{n}$ is the mean of the ROM for each joint all control group subjects, and ${\alpha }_{w}$ is the mean of $ROM$ for two cases: Re-fill with P/S and Re-fill without P/S.

#### Statistics

For motion analysis, we calculated the average joint angle for each joint measured during the reach-to-grasp task using all trials and subjects in the control group. We also compared the muscle synergy with the control group to patients with P/S and patients without P/S. We prepared the data for statistical analysis using repeated the reach-to-grasp task (4 sets of 6 trials each set, totaling 24 trials). For the group of 10 subjects, we gathered data as follows: (10 subjects × 4 sets × 6 trials)/10 subjects = 24 trials. We analyzed the statistics between groups, taking into account the sample size of each group's dataset (control group N = 24 trials, with P/S N = 24 trials, and without P/S N = 24 trials).

The Wilcoxon rank-sum test was applied to confirm the differences between the control group 10 healthy subjects and the partial hand amputee with P/S, the control group and the partial hand amputee without P/S, and the cases with and without P/S. This was done using non-parametric tests. The Kruskal–Wallis test was applied to confirm the difference between the control group, the group with P/S, and the group without P/S. In addition, to determine the effect of the wrist rotation module on the amputees' upper limb movement, the CM ratio was calculated, and a non-parametric Wilcoxon rank-sum test was used for the with P/S and without P/S cases.

For the muscle synergy analysis, a partial hand amputee, both with and without P/S, was compared to a control group of 10 healthy subjects who participated in the study. The number of muscle synergies, as determined by VAF, was set based on a threshold (gVAF > 90%, mVAF > 75). When the number of muscle synergies was smae, the synergy vectors derived from NMF were compared. However, if the number of muscle synergies varied, the groups were considered distinct and thus were not compared. The statistical significance between each group (control, amputee with P/S, and amputee without P/S) was then verified. The Wilcoxon rank-sum test was employed for each muscle synergy vector to compare each muscle between the control and an amputee. All data comparisons were performed using MATLAB software (MATLAB 2021a Math; MathWorks Inc., MA, USA). Using statistical parametric mapping, the activation patterns were compared between the two groups (control vs. with P/S). Statistical significance was set at a p-value < 0.05.

## Results

### JHFT score

**Fig. 5:**
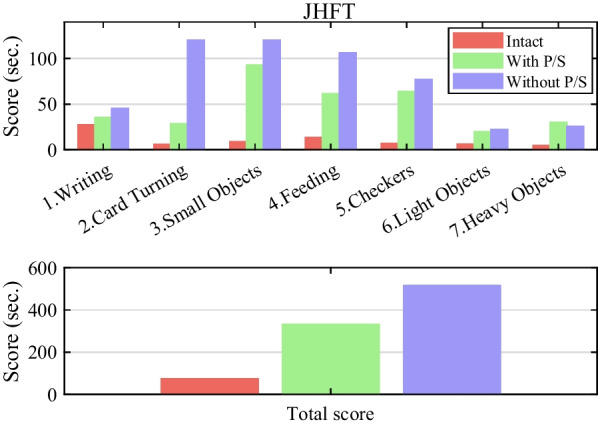
The subset and total score of Jebsen-Tyler hand function test

The overall result score of JHFT is shown in Table 3 and Fig. 5. The total score varied from 516.37 s without P/S to 331.46 s with P/S, and this result showed that the wrist-rotation module could improve the subject’s ADL performance (Table 3). The total JHFT scores were 37.9 s for the normalized JHFT and 73.33 s for the intact side. For subsets 1,2,4,5 and 6, the with P/S was faster on the amputated side. In contrast, subsets 3 and 7 were measured to be faster in the case without P/S. However, the cases with and without P/S showed a similar difference. Specifically, if there was a wrist rotation module, it took longer to complete a subset than on the intact side.

**Table 3:** Score of Jebsen-Tyler hand function test

JHFT subsets	Intact side (left)	Amp.side -with P/S	Amp.side -without P/S	Standard (D/N) [70]
1. Writing	27.25	35.31	45.22	12.3 (32.3)
2. Card turning	5.88	28.37	120	4.0 (4.5)
3. Small common objects	8.82	92.87	120	5.9 (7.9)
4. Simulated feeding	13.38	61.22	106.37	6.4 (7.9)
5. Checkers	6.88	63.81	76.97	3.3 (3.8)
6. Large light objects	6.19	19.88	22.31	3.0 (3.2)
7. Large heavy objects	4.93	30.00	25.50	3.0 (3.1)
Total (s)	73.33	331.46	516.37	37.9 (62.7)

*Amp.* amputated side, *D* dominant, *N* non-dominant

### Reach-to-grasp task test

#### Motion analysis

The object's trajectory was not statistically significant when the reach-to-grasp task was completed from start to finish (Table 4). In Table 4, when comparing the control and P/S groups, there was a statistically significant difference in wrist flexion/extension (p = 0.042) and no statistical significance in the remaining joint angles. In the control and without-P/S groups, there were statistically significant differences in shoulder abduction and adduction (p = 0.001), internal/external rotation (p = 0.048), and wrist pronation/supination (p = 0.001). There was a statistically significant difference between with and without P/S in shoulder abduction/adduction (p = 0.001), internal/external rotation, wrist flexion (p = 0.001), wrist flexion/extension (p = 0.046), and wrist pronation/supination (p = 0.004). In three cases, trunk flexion/extension, shoulder abduction/adduction, shoulder rotation, wrist flexion/extension, and wrist pronation/supination were statistically significant (p < 0.05).

**Table 4:** Motion analysis during the reach-to-grasp task

Kinematic variable	Control	With P/S	Without P/S	Control vs with P/S	Control vs without P/S	With P/S vs without P/S	Control vs. with P/S vs. without P/S
	Mean (SD)			P-value			
Object trajectory (mm)	489.74 (4.46)	527.70 (8.39)	552.39 (11.05)	0.057	0.061	0.450	0.091
*Trunk angle (deg.)*							
Flexion/extension	0.15 (0.14)	− 0.9 (0.36)	3.27 (1.35)	0.068	0.032	0.020	0.041*
Rotation	− 2.71 (1.88)	− 3.55(1.94)	− 3.78 (1.85)	0.178	0.067	0.097	0.078
*Shoulder angle (deg.)*							
Flexion/extension	21.19 (15.16)	24.43 (11.47)	29.58 (14.34)	0.054	0.052	0.062	0.0613
Abduction/adduction	10.72 (7.68)	10.21 (6.44)	34.26 (15.27)	0.540	0.001*	0.001*	0.001*
In./external rotation	7.74 (4.68)	9.55 (4.22)	32.20 (14.08)	0.068	0.048*	0.001*	0.000*
*Elbow angle (deg.)*							
Flexion/extension	− 13.75 (8.52)	− 20.80 (7.00)	− 21.69 (8.67)	0.059	0.061	0.087	0.058
*Wrist angle (deg.)*							
Flexion/extension	28.95 (14.43)	38.13 (13.50)	20.50 (7.49)	0.042*	0.056	0.046*	0.037*
Supination/pronation	72.40 (37.43)	76.53 (24.91)	34.09 (12.25)	0.169	0.001*	0.004*	0.002*

**Fig. 6:**
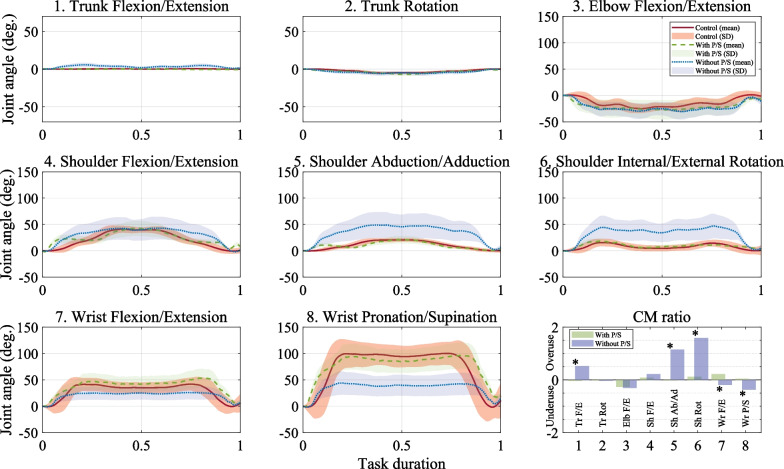
A graph of the angles of all joints during reach-to-grasp. Bold lines: mean joint angle of the control group. Shaded part: SD of each joint angle of the control group, broken line: mean joint angle of the Re-fill with P/S, dotted line: mean joint angles of the Re-fill without P/S

We used an asterisk (*) to denote the significant difference. The statistically significant difference between the 'with P/S' and 'without P/S' conditions for each joint was verified using the CM ratio. The CM ratio is shown in Fig. 6. Statistically significant differences (p < 0.05) were observed in wrist pronation/supination, shoulder rotation, and abduction/adduction, also, wrist flexion/extension and trunk flexion (p < 0.01). In the Fig. 6, a CM ratio of 0 is interpreted as being equivalent to the level of a healthy individual. A positive value indicates an overuse of movement in the joint compared to a healthy person, representing compensatory movement. Conversely, a negative value signifies movement less than that of a healthy individual, indicating underuse.

#### Muscle synergy analysis

**Fig. 7:**
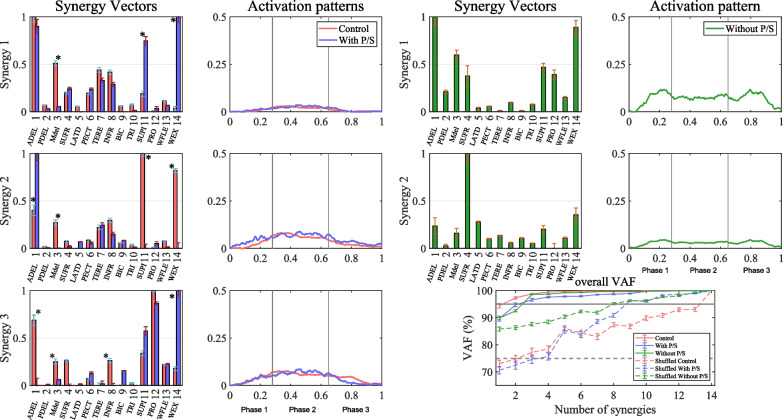
The muscle synergy vectors and the synergy activation patterns. The left side displayed the control group, and with P/S case, the right side displayed the without P/S case, and the bottom side showed VAF

The number of muscle synergies was selected when the gVAF was over 90%, and mVAF was over 75%. The same number of synergy patterns (N = 3) was observed in both the control group and the amputee with P/S. Therefore, we compared the synergy vector of muscle synergy between the control group and the amputee with P/S. The amputee without P/S had only two synergy patterns. As a result, we could not compare the amputee without P/S. In Fig. 7, three types of muscle synergy vectors and their corresponding activation patterns are shown.

We obtained the results of muscle synergy analysis in terms of synergy vectors and activation patterns for the control group and the amputee with and without P/S. The result was shown for both the control group and the amputee with P/S, both having the same number of synergies (N = 3). We used an asterisk (*) to denote the significant difference in Fig. 7. Synergy 1 of activation patterns was observed in the transport and release phase (Phase 2) for both the control group and an amputee with P/S. The synergy vector was significantly different (p < 0.05) in the middle deltoid (3 MDEL), supinator (11 SUPI), and wrist extensor (14 WEX) muscles. In the control group and with P/S, synergy 2 of activation patterns shown in grasp and return Phase 1 and Phase 2, and the synergy vector showed statistically significant differences (p < 0.05) in the anterior deltoid (1 ADEL), middle deltoid (3 MDEL), and supinator (11 SUPI). Synergy 3 of activation patterns was observed in Phase 2 and 3, and the synergy vector showed significant differences (p < 0.05) in the anterior deltoid (1 ADEL), middle deltoid (3 MDEL), infraspinatus (8 INFR), and pronator (12 PRO). There was no statistically significant difference between the control and with P/S groups in the muscle activation patterns of synergies 1, 2, and 3.

The amputee without P/S had two muscle synergies. Synergy 1 was characterized by high usage of the anterior deltoid (1 ADEL), wrist extension (14 WEX), and middle deltoid (3MDEL), whereas synergy 2 tended to rely most heavily on the supraspinatus (4 SUFR).

## Discussion and conclusion

This study proposes a wrist rotation module prototype that can be applied to hand amputation. To verify this, we compared upper limb muscle synergy and movement patterns with a control group to evaluate the quality of upper limb movement during the reach-to-grasp task. As a result, it was confirmed that the natural muscle synergy pattern of the control group was recovered when wrist rotation was allowed in a partial hand amputee with limited wrist movement using the prototype of the proposed wrist rotation module. Because of this, the movement pattern became identical to that of the typical strategy, and it was found that there was a difference in the compensatory movement compared to the case where the wrist rotation module was not used. In addition, the JHFT results indicated that upper limb movement pattern-based hand function exhibited sustained benefits for the entire time. Natural muscle synergies can be restored by applying rotational wrist movements. In conclusion, if limited wrist rotation is considered when designing a prosthetic hand for partial hand amputation, natural muscle synergy should be restored to offer limited compensatory movement and improve usability by preventing secondary musculoskeletal damage.

### The proposed design of the wrist rotation module

The proposed wrist rotation module prototype was designed to satisfy the requirements for wrist rotational movement. It also enables wrist-movement assistance without interfering with hand movements. It is lighter than the manufactured wrist prosthesis (600–800 g) as mentioned in previous studies [14, 31, 35], and can be applied to daily life. In addition, the socket design and wearing method are convenient for increasing usability.

Traditionally, the upper limb prostheses have primarily focused on terminal devices such as hands or grippers, often overlooking the significance of the wrist [11, 39]. However, recent prosthetic studies [28, 31] have revealed that the skillfulness of the wrist can greatly influence manipulative ability, surpassing highly proficient terminal devices with limited wrist function. The importance of the wrist becomes particularly evident in tasks involving a cylindrical grip or when a simple design end effector is utilized, and the fingers are fully constrained [28]. These findings highlight the growing recognition of the wrist's role in the evolving design of prosthetics, with an emphasis on simplicity, including lightweight construction, cost-effectiveness, and fatigue reduction [36].

Although not a partial hand amputee, there are prosthetic wrists that allow wrist rotation for trans-radial or trans-humeral amputation [15, 34, 57]. In [31, 41], it was the passive single-DOF wrist prosthesis by OttoBock, known for its mechanical simplicity and lightweight design. However, manual operation poses inconvenience. To address this, actuation-type prosthetic wrists have been developed [31]. Another common approach is using active rotators, such as electric rotation devices like TB i-limb Quantum [31]. While they offer improved maneuverability and reduced the prosthetic system length, they still cannot apply for partial hand amputees. To overcome these limitations, we proposed the DPM for a person with partial hand amputation that offers a lightweight and compact solution using a four-bar linkage structure, delivering comparable power of the wrist rotation.

In this study, our aim was to investigate the joint movement and muscle synergistic effects of the entire upper limb when wrist rotation was allowed. When prosthetic hand users control their prosthesis using muscle signals, the overlap between control and movement signals makes it difficult to distinguish distinct muscle synergies. Therefore, we did not adopt myoelectric control. Through our experimental design for muscle synergy, we observed that incorporating a wrist rotation design restored normal levels of muscle synergy and improved upper limb kinematics. However, prosthetic operation methods remain a primary focus, and various approaches, such as electrical motor methods and machine learning method using muscle signal, have been explored. Although advancements in myoelectric control techniques have been significant [72], challenges persist due to signal variability and mismatching with prosthetic hand/arm DOF, necessitating long-term training for adaptation. Recent studies have investigated muscle synergy information [73] and residual upper limb movement trajectories [74] to address these challenges. Our study results indicate the potential to develop prosthetic control technology that enables natural, convenient movement while preventing secondary damage, by leveraging muscle synergy and the remaining upper limb trajectory.

### JHFT

The JHFT results showed that the intact side of the amputee had the highest score compared to the amputated side, but it took longer when measured using a prosthetic hand. However, in the case of the with P/S, better results were obtained based on the final scores. When evaluating hand function with a prosthetic hand that incorporates a wrist rotation module, the benefit of usage time can be obtained in terms of usability.

The four subsets of JHFT [2, 3, 6, 7] necessitate wrist rotation, which emphasizes its essential role in completing functional movements of the hand. When the test was conducted while maintaining a sitting posture to provide a consistent experimental environment, the experimental results of items 2 and 3 of the JHFT were impossible to perform without wrist rotation. This can be seen as not being resolved by shoulder rotation alone. In addition, in items 6 and 7 of the JHFT, in which the object was lifted and moved, the compensatory movement of the shoulder rather than the trunk was overused because the object's size was relatively large compared to other items [71]. In other words, it was confirmed that the compensation pattern could be significantly affected by the object size [43, 50]. The JHFT is an easy-to-use method for evaluating hand function, and it is possible to assess the primary hand function necessary for ADL. The JHFT provides typical scores for each item's dominant and non-dominant hands (Table 3). On the intact side, it appeared at a similar level to the typical JHFT scores. However, typical JHFT scores differed when the prosthetic hand (with and without P/S) was worn. This is because performing JHFT with a prosthetic hand may cause further usage delays (of 1–2 s) since the movement of the prosthetic hand depends solely on visual feedback. This indicates that improving the sensory feedback system is necessary to achieve high usability [50]. Nevertheless, using the wrist rotation module improved usage time, and feasible tasks related to hand function completion increased.

### Motion analysis

We compared the control group and an amputee with or without P/S during the reach-to-grasp motion: trunk flexion/extension, rotation, shoulder flexion/ extension, shoulder abduction/adduction, shoulder internal/ external rotation, wrist flexion/extension, and wrist pronation supination (Table 4). There were statistically significant differences in trunk flexion, shoulder abduction, internal rotation, and wrist flexion and pronation, but not in trunk rotation, shoulder flexion/extension, and elbow flexion/extension.

A statistically significant difference was observed in wrist flexion in the amputee with P/S. When the tip of the prosthetic hand with P/S touched the floor, more wrist flexion was performed to control the tip of the pronated hand prosthesis. Compared to the control group the amputee showed a lower mean value in wrist flexion but no statistically significant difference, whereas a statistically significant difference was observed in wrist pronation. Shoulder abduction, internal rotation, and trunk flexion were used differently to the extent that there was a statistically significant difference in grasping an object using a prosthetic hand. This was because a compensation pattern strategy using the shoulder and trunk was used, and the wrist's movement was relatively small. A previous study reported that shoulder joint use increased when wrist joint movements were limited [28, 31].

The wrist and shoulder provide compensation strategies for each other, which we have shown in the CM ratios. Shoulder abduction, internal rotation, and trunk flexion are overused when wrist pronation is underused. This indicates an inefficient upper-limb movement pattern in which the movement of the shoulder with two DOFs is used to compensate for the lack of one DOF in the wrist. Wrist pronation rotates around the third finger and draws a circle around the axis of rotation of the first finger. The position near the first finger was adjusted by orienting it as far away as the radius from the center of the rotation axis. Adjustments in the front and rear orientations of the hand occur on the trunk connected to the shoulder. It is a compensatory pattern that deviates from the natural movement pattern, in which the entire arm is moved to adjust the hand's orientation. Overuse of the shoulder joint, which is larger and heavier than the wrist, learns an inefficient movement pattern that can cause secondary injury and pain. As mentioned in other studies [41, 42, 49], amputees often use movement strategies that achieve their goals with compensation patterns using upper-extremity joints other than the amputation site. As such, the wrist can achieve its final purpose with a cooperative relationship between the upper limb and body; therefore, engineering and technology should be considered in the wrist part to design the prosthetic hand.

### Muscle synergy

This study is the first to investigate differences in amputees' trunk and upper limb muscle synergy compared with healthy subjects while using a prosthetic hand of the amputee. The main purpose of the upper limb is to enable the hand as an end effector to reach its final goal efficiently without damage, working in cooperation with various joints in the upper limb. The musculoskeletal system with multiple DOF generates a natural movement pattern optimally using muscle synergy to achieve this purpose. However, limited rotational movement of the wrist causes changes in muscle synergy, resulting in alterations in movement patterns.

Our analysis showed that when the partial hand amputees were allowed a limited DOF of wrist rotation through the prosthetic hand, the number of muscle synergies was the same as that of the control group. Most studies [48, 59] analyzed limb movements using kinematic changes to evaluate the usability or performance of prosthetic hands. However, changes in the kinematic outcomes result from multiple distinct neuromuscular strategies with different muscle activation patterns. The muscle synergy analysis can be a valuable metric for movement performance level through changes in muscle synergy number, weight vectors, and muscle activation patterns. For instance, the number of muscle synergies between a skilled expert and a first-time performer during the same task is higher for the expert, which can be interpreted as being able to control the movement finely. In other words, allowing wrist rotation motion in partial hand amputees indicates that natural movement strategies can be recovered by restoring the same muscle synergies as healthy people during the reach-to-grasp task. When muscle synergy was observed in stroke subjects in previous studies [58, 71], it was confirmed that they had lower muscle synergy numbers than control group. This could be interpreted as an instability factor, in which a lower number of muscle synergies can decrease the accuracy of upper-limb movement and expand the workspace during upper-limb movement. It has been reported that the limitation of actual wrist movement expands the direction of the shoulder joint, which requires more workspace [75]. Therefore, to recover the natural movement pattern and muscle synergy strategy, the rotational movement of the wrist should be considered when designing a prosthetic hand.

The number of muscle synergies in prosthetic users who have access to a wrist rotation module could match that of the control group with healthy subjects. This is because the gross motor skills of large muscles such as the trunk and arm (shoulder and elbow) are learned naturally through typical motor development and are quickly recovered even after a long period of non-use [76]. However, the neuromuscular system is related to the motor control strategy of the central nervous system, and sensory feedback can be significant. In the future, it will be necessary to connect the sensory feedback system of the prosthetic hand to detect changes in movement patterns.

On the other hand, the number of muscle synergies was the same as that of the control group with healthy subjects, but there was some difference in the weight vector. In particular, the use of wrist extension was high, and the frequency of shoulder flexion increased in the control group. This is due to the minimal damage to the wrist extension muscles in the amputee, and the burden of constantly enduring the weight of the prosthetic hand. However, the muscle synergy, weight vector, and muscle activation patterns were different when using a prosthetic hand without wrist rotation. This can be interpreted as an altered neuromuscular strategy to compensate for the lack of wrist rotational movement. In addition, the shoulder's supraspinatus, and middle deltoid muscles, which are often used during the task, overlap with the muscles that cause impingement syndrome [77]. This can be interpreted as the increased risk of secondary musculoskeletal damage when an amputee uses a prosthetic hand without wrist rotation as a continuous compensatory pattern. In [78], a difference in the muscle synergy vector was reported when the muscle synergy was investigated between swimmers with and without shoulder pain.

This study focus on obtaining representative synergies of natural movements from 10 healthy individual and determining the degree of restore by comparing these with amputees, and it was confirmed that they showed similar results in terms of the number of muscle synergies. Nevertheless, it was observed that the synergy vectors of synergy 2 and 3 did not show similar tendencies. This discrepancy is due to the physical differences caused by the amputees' lost joints and damaged muscles. In previous studies [43], muscle synergies have identified differences between amputees and able-bodied individuals while performing a reaching task. They reported that, during a reaching task, amputees experienced difficulties in preliminary postural control before final task time due to the lack of sensory feedback, leading to differences compared to able-bodied individuals. In other studies [79, 80] that focused on subject-specific muscle synergies, it has been reported that various task conditions, such as changes in the direction or speed of the task, can influence muscle synergies.

In the case of prosthesis control, there is much interest in the bio-signals, such as sEMG signal, that are utilized in how the prosthetic hand works [42]. Measurements of neurophysiological signals, such as sEMG activity, provide a comprehensive characterization of motor control and valuable insights into motor control strategies [10]. However, owing to the large number of muscles and joints in the human body, it is difficult to control their application in robots. Several studies have reported that healthy people can control a robot using a muscle synergy strategy, and long-term robot control is achievable with this approach. However, this did not apply to amputees, and the muscle synergy pattern of the amputees could not be guaranteed. We investigated the muscle synergy of the amputee and found a level of muscle synergy similar to that of healthy individuals when rotational movements were allowed. Our results showed that partial hand amputees might use muscle synergy to control the prosthetic hand if wrist rotation is permitted.

### Limitations

The limitation of this study is that the wrist rotation module operation method is on/off, so improvement is required. The task of upper-limb movement needs to be investigated in various settings. Muscle synergy can be observed in the fact that the use of muscles may vary owing to the absence of sensory feedback. Therefore, in future research, it will be necessary to investigate the changes in the pattern of muscle synergy by adding sensory feedback.

This study compared one amputee with a control group. However, we could not compare the effect on synergies due to the with or without of a wrist rotation module (P/S) in the same partial hand amputee. Therefore, there is a need to recruit more amputees to compare the effects of the with or without of a wrist rotation module applied to amputees.

Prosthetic engineers play a crucial role in fostering user awareness and improving the technical aspects of prosthetic devices. It is essential for prosthetic device users to recognize the importance of various usability attributes. These attributes extend beyond the functional performance of the device and encompass aspects such as facilitating natural movement and complying with proper body mechanics during operation, as highlighted in this study. Additionally, ensuring ease of use necessitates streamlined initial setup processes and regular maintenance. Moving forward, this research will delve into the design and methodology considerations, particularly focusing on the usability of prosthetic hands, with a strong emphasis on enabling natural movement and optimal body mechanics.

## Data Availability

Data and materials can be made available upon request to the authors.
